# Enhancing the Seebeck effect in Ge/Si through the combination of interfacial design features

**DOI:** 10.1038/s41598-019-52654-z

**Published:** 2019-11-08

**Authors:** Andriy Nadtochiy, Vasyl Kuryliuk, Viktor Strelchuk, Oleg Korotchenkov, Pei-Wen Li, Sheng-Wei Lee

**Affiliations:** 1 Taras Shevchenko Kyiv National University, Department of Physics, Kyiv, 01601 Ukraine; 2 V.E. Lashkaryov Institute of Semiconductor Physics, National Academy of Sciences of Ukraine, Kyiv, 03028 Ukraine; 3 National Chiao Tung University, Department and Institute of Electronics Engineering, Hsinchu, Taiwan 30010 Republic of China; 4 National Central University, Institute of Materials Science and Engineering, Jhongli, 32001 Taiwan Republic of China

**Keywords:** Thermoelectric devices and materials, Quantum dots

## Abstract

Due to their inherent physical properties, thin-film Si/SiGe heterostructures have specific thermal management applications in advanced integrated circuits and this in turn is essential not only to prevent a high local temperature and overheat inside the circuit, but also generate electricity through the Seebeck effect. Here, we were able to enhance the Seebeck effect in the germanium composite quantum dots (CQDs) embedded in silicon by increasing the number of thin silicon layers inside the dot (multi-fold CQD material). The Seebeck effect in the CQD structures and multi-layer boron atomic layer-doped SiGe epitaxial films was studied experimentally at temperatures in the range from 50 to 300 K and detailed calculations for the Seebeck coefficient employing different scattering mechanisms were made. Our results show that the Seebeck coefficient is enhanced up to ≈40% in a 3-fold CQD material with respect to 2-fold Ge/Si CQDs. This enhancement was precisely modeled by taking into account the scattering of phonons by inner boundaries and the carrier filtering by the CQD inclusions. Our model is also able to reproduce the observed temperature dependence of the Seebeck coefficient in the B atomic layer-doped SiGe fairly well. We expect that the phonon scattering techniques developed here could significantly improve the thermoelectric performance of Ge/Si materials through further optimization of the layer stacks inside the quantum dot and of the dopant concentrations.

## Introduction

Due to dense packaging in high power electronics, the heat generation in chips can reach ~50 W/cm^2^, which produces uneven temperature distributions with 5 °C to 30 °C overheated hot spots and decreases the reliability of silicon-based electronic components^1–3^. These hot spots can generate electricity through the Seebeck effect by harvesting waste heat from electronic circuits. Through the reverse Peltier effect, on-chip cooling has been achieved in thin-film Si/SiGe superlattice micro-refrigerators, which allows effective on-chip temperature control^3^.

**Figure 1:**
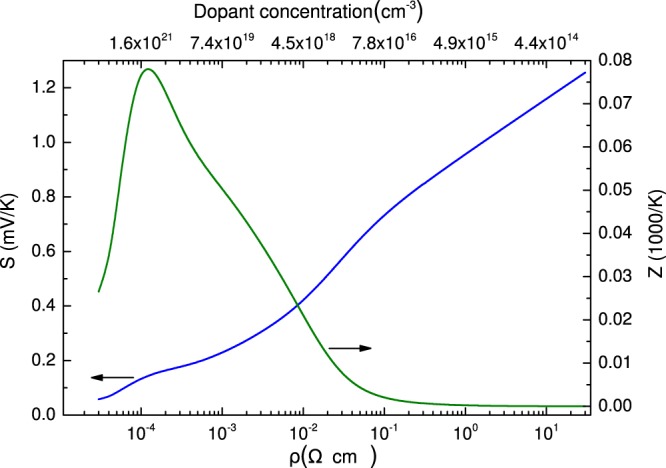
Variation of the Seebeck coefficient and figure of merit upon electrical resistivity for a bulk Si at *T* = 300 K. Theoretical estimates are made using the works of Yi and Yu^47^ and Morelli *et al*.^59^. Relaxation time *τ*_*p*_ is obtained by taking into account phonon-phonon and Umklapp scattering, phonon-alloy and boundary scattering. *τ*_*e*_ takes into account electron scattering by impurities and phonons.

The thermoelectric efficiency is most conveniently determined by the figure of merit, *Z* = *S*^2^/*κρ*, where *S* is the Seebeck coefficient, *κ* is the thermal conductivity and *ρ* is the electrical resistivity. Therefore, the better thermoelectric performance can be obtained at greater *Z*, requiring greater *S* and smaller *κ* and *ρ*. The search for efficient thermoelectric materials is challenging due to the multitude of conflicting property requirements that must be simultaneously satisfied, which is because the coefficients *S*, *ρ* and *κ* are usually related to one another and are not mutually exclusive^4^. Figure 1 compares the variation of *S* and *Z* as a function of the electrical resistivity. It is seen that increasing *ρ* enhances the Seebeck coefficient while much smaller optimal electrical resistivities, corresponding to the dopant concentrations of about 10^20^–10^21^ cm^−3^ in Fig. 1, maximizes the figure of merit.

Improving Seebeck coefficient is very relevant to numerous applications utilizing thin-film approach to achieve thermoelectric sensing functionality^2,5^. One common strategy for increasing *S* relies on the enhancement of the phonon scattering, which is ultimately governed by interfaces and boundaries in nanostructured materials^2,6–8^.

In particular, Choi *et al*. reported thermoelectric Te-embedded Bi_2_Te_3_ materials with enhanced scattering of phonons at the inter-grain Te/Bi_2_Te_3_ boundaries^9^. Ahmad *et al*. designed nanocomposite SiGe-TiO_2_ materials with superior thermoelectric properties due to TiO_2_ inclusions with coherent interfaces and modulation dopant regions of boron^10^. This finding of the intentional doping as a new design of thermoelectric devices has some implications on a study performed by Zardo and Rurali using the strategy of varying impurity concentrations to tune the amount of phonon scattering events^11^. The evidence for the fact that the scattering of phonons by nano-scale grains and nanotwins in InSb samples can straightforwardly be related to their thermoelectric behaviour came from the work of Mao *et al*.^12^. With this in mind, the Seebeck coefficient can be increased by the filtering of low energy charge carries at the interfaces, as pointed out by Zhao *et al*.^13^. It was further shown by Yadav *et al*. that the thermoelectric properties of composite materials can be improved by adding different two-dimensional components in the host matrix^14^. Further progress was achieved very recently by Sakane *et al*. owing to fabrication of Si films with various nanodots and atomic-scale impurities, which act as scattering centers controlling the carrier transport^15^. This insight was improved considerably by fabricating textured polycrystals of superlattice phases, which generate appropriate potential barriers that can manipulate the phonon and electron transport^16^. Furthermore, as shown by Kim *et al*., the inclusion of the conducting polymer, polypyrrole, in the Bi_2_Te_3_ matrix enhances the phonon scattering at the interface of the two components in this hybrid material and thus makes the Seebeck coefficient considerably greater than one achieved in pristine Bi_2_Te_3_^17^. As noted by Tureson *et al*., the presence of Mg implanted ions in epitaxial ScN films introduces an interesting approach to enhance the Seebeck coefficient^18^. Tayari *et al*. found high Seebeck coefficient in a quasi-two-dimensional semiconducting material, such as layered SnSe^19^.

Chang *et al*. previously introduced Ge/Si composite quantum dots (CQDs) assembled on Si. which can effectively scatter phonons due to Si sub-dot stacks formed inside the dot, thereby reducing *κ* in the composites^20,21^.

In order to construct a mathematical model for the thermoelectric voltage, it is useful to consider (i) the difference in work function, *S*_*φ*_, between the electrode and semiconducting materials, which is important in solid state thermionic applications, (ii) the diffusive transport of charge carriers across a temperature gradient, *S*_*d*_, and (iii) the phonon drag component, *S*_*p*_, which arises from electron drag by phonons, so that *S* = *S*_*φ*_ + *S*_*d*_ + *S*_*p*_^22–24^. Typically, *S*_*d*_ is the most dominant component, while *S*_*p*_ is frequently taken into account in doped samples, especially at low temperature^6^. Thus, it was previously suggested by Boukai *et al*. that increase in *S* observed in Si nanowires is due to increased *S*_*p*_^25^.

The *S*_*p*_ component can be estimated as^26^

$${S}_{p}=\frac{\rho ne\beta {\upsilon }_{p}{l}_{p}}{T},$$

where *n* is the carrier concentration, *e* is the elementary charge, 0 < *β* ≤ 1 is the strength of the electron-phonon interaction, *υ*_*p*_ is the phonon velocity and *l*_*p*_ is the phonon mean free path. In calculating *l*_*p*_, we take into account only phonons participating in the phonon-mediated drag effect. Therefore, *l*_*p*_ in fact greatly exceeds the mean free path of an average phonon, since the low-wavenumber vibration modes mainly interact with the electrons. Consequently, the *S*_*p*_ component is normally small for high doping, while at low doping it can dominate over the *S*_*d*_ component, as was indeed observed in Si by Weber and Gmelin^26^.

The main advances in the understanding of these thermoelectric properties came from the Boltzmann transport theory^23,27,28^. In particular, taking into account the scattering of phonons at boundaries increases *S* due to filtering or quantum confinement of free carriers^29–31^. Most recently, Vargiamidis *et al*. modeled the Seebeck coefficient in superlattice materials taking the relaxation processes in the barrier and well regions^32^ as well as the thermoelectric behaviour of hierarchically nanostructured materials employing the nonequilibrium Green’s function technique^33^. Phonon relaxation times in aperiodic polycrystalline nanostructures were approximated by Ohnishi and Shiomi^34^. The phonon transport and confinement in the layered structural blocks were addressed by Fiorentini *et al*. utilizing *ab initio* anharmonic computations^35^. Gelda *et al*. estimated phonon lifetimes from the scattering theory at rough surfaces, which are suitable to describe the properties of thermal transport in nanostructures^36^.

Here, we attempt to realize a new strategy for enhancing the Seebeck coefficient by optimizing an interfacial design in Ge/Si nanostructures. This was done by employing multi-fold Ge/Si composite quantum dots with thin silicon layers placed inside the germanium quantum dot. Our experiments revealed that this CQD material can offer significantly enhanced Seebeck effect. The observed enhancement was modeled by precisely taking into account the scattering of phonons by inner boundaries and the carrier filtering by the CQD inclusions. The applicability of such a modeling approach was independently verified by using GeSi films with boron atomic layer doping that allow precise control of the phonon scattering. We therefore provide a direct experimental and computational evidence that using the composite Ge/Si quantum dot inclusions provides an effective path to enhance the Seebeck coefficient. In order to accurately quantify the contribution of the phonon-interface scattering in the composite quantum dots and to surpass the likely involvement of the impurity scattering effects, undoped CQD layers were employed here. Varying the dopant concentration of each layer is thought to have a preponderant effect on the resultant high-performance thermoelectric behaviour.

## Samples and Thermoelectric Measurement Techniques

Three sample sets were analyzed. The samples of set L were obtained depositing a 100 nm thick Ge_*x*_Si_1−*x*_ alloy layer on a p-doped (001) single silicon-on-insulator (SOI) wafer, which comprised 55 nm Si, 150 nm SiO_2_ buffer and 500 *μ*m Si substrate. The thickness of the device Si layer (10 Ω × cm) was reduced to about 10 nm by wet oxidation and HF etching. Ge_*x*_Si_1−*x*_ films were deposited at 500 °C by chemical vapor deposition (CVD) using SiH_4_/GeH_4_/H_2_ with the gas pressure of 200 Pa. The concentration of Ge in the grown SiGe films was about 30%, the resistivity was 0.03 Ω × cm, resulting from the B doping level of about 10^18^ cm^−3^. This sample set was used to judge the reliability of our Seebeck measurements and calculations.

The second set MDL was made using atomic-level control of B doping in a 450 nm thick Ge_*x*_Si_1−*x*_ film, following the methodology described elsewhere^37^. Epitaxial growth of multi-layer B-doped Ge_*x*_Si_1−*x*_ was carried out by alternately supplied B_2_H_6_ and SiH_4_/GeH_4_ in H_2_. In this case, the growth of the base Ge_*x*_Si_1−*x*_ material was terminated and the temperature was set at 400 °C. During this growth interruption, the sample surface was exposed to B_2_H_6_. Thereafter, the Ge_*x*_Si_1−*x*_ growth was continued. The Ge content in the resulting Ge_*x*_Si_1−*x*_ films was about 25%, the resistivity was 0.04 Ω × cm. Typical secondary ion mass spectroscopy (SIMS) profile of the boron concentration is shown in Supplementary Fig. S1.

**Figure 2:**
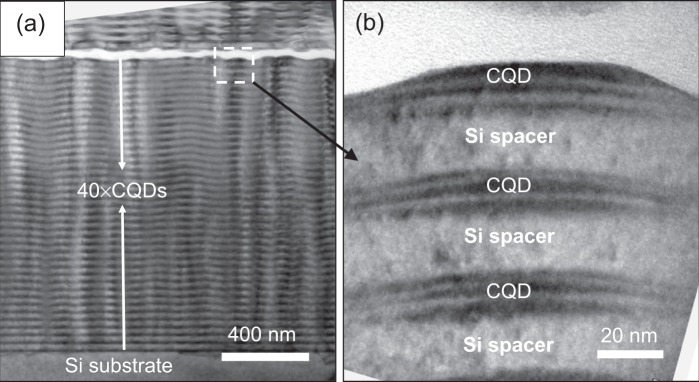
XTEM micrograph of a 3-fold coupled QD structure (40 periods of CQD/Si stacks) (**a**). A magnified image of the dashed rectangular region in (**a**) for three selected CQDs near the top stack is shown in (**b**). Each CDQ is composed of Ge layers (three distinct dark regions shown in the right-hand image), whereas the two white areas corresponding to Si layers are inserted into the dot. Higher resolution image of CDQs is given in Supplementary Fig. S2.

A further sample set was a series of multifold Ge/Si/Ge stacked structures (CQDs) with varying thermoelectric functionality, which is due to different thicknesses of inserted Si and numbers of Ge stacks. They were CVD grown on *p*-type (001)-oriented Si wafers (10–25 Ω × cm), 150 mm in diameter, with ≈0.15 nm thick insulating layer of SiO_2_, which was used to electrically isolate the stacks from the substrate. The growth temperature was set at 600 °C. Further details on the structure growth system can be found elsewhere^20,21^. Here, two sets of samples were compared, which are referred to as 2-fold and 3-fold CQDs. The 2-fold CQDs consists of the sequence of Ge (12.6 MLs)/Si (2 nm)/Ge (12.6 MLs) layers, whereas the Ge (12.6 MLs)/Si (2 nm)/Ge (12.6 MLs)/Si (2 nm)/Ge (12.6 MLs) sequence was developed in 3-fold CQDs. As shown in Fig. 2(a), 40-period multifold CQDs were grown to build up a thin-film-like material, ~1.5 *μ*m thick, for thermoelectric measurements. Each multifold CQD layer was separated by 20 nm Si spacer layers.

Although non intentionally doped, those CQD fllms revealed *n*-type behavior with a room-temperature resistivity of about 0.09 Ω × cm, which corresponds to concentrations of ≈1 × 10^17^ cm^−3^. This may be due to the fact that type II Ge/Si heterostructure forms the potential well for holes in the valence band of Ge_*x*_Si_1−*x*_ inclusions^38^. Our theoretical estimates gave the resulting electron concentration of ≈0.5 × 10^17^ cm^−3^ in the CQD fllms, which is fairly close to the measured value.

**Figure 3:**
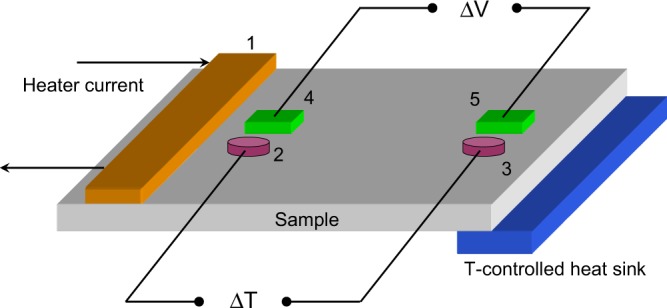
A schematic diagram for the Seebeck coefficient measurement. 1 - heater (100-Ω SMD resistor), 2 and 3 - temperature sensors (BAP64-02NXP diodes), 4 and 5 - metal pads (InGa), which form Ohmic contacts to the semiconductor. Heat is extracted by channeling it into the cold head of the cryostat connected directly to the sample edge (*T*-controlled heat sink).

The Seebeck coefficient was measured using the general approach reviewed by Martin *et al*.^39^. Our automatic home-built measuring system is schematically sketched in Fig. 3. Because of flow of current through the heater with a resistance *R* (1 in Fig. 3) mounted on the sample the temperature rises at the heater side of the sample. A thermal bath at the opposite sample side works as a temperature-controlled heat sink (Fig. 3). In turn, a local temperature difference Δ*T* = *T*_2_ − *T*_3_ is built up between temperature-sensing diodes 2 and 3, and the thermoelectric voltage Δ*V* is measured between contacts 4 and 5. The temperature sensors were calibrated before Seebeck measurement. A closed-cycle cryostat (CS204, Advanced Research Systems) was used to vary the temperature of the sample.

**Figure 4:**
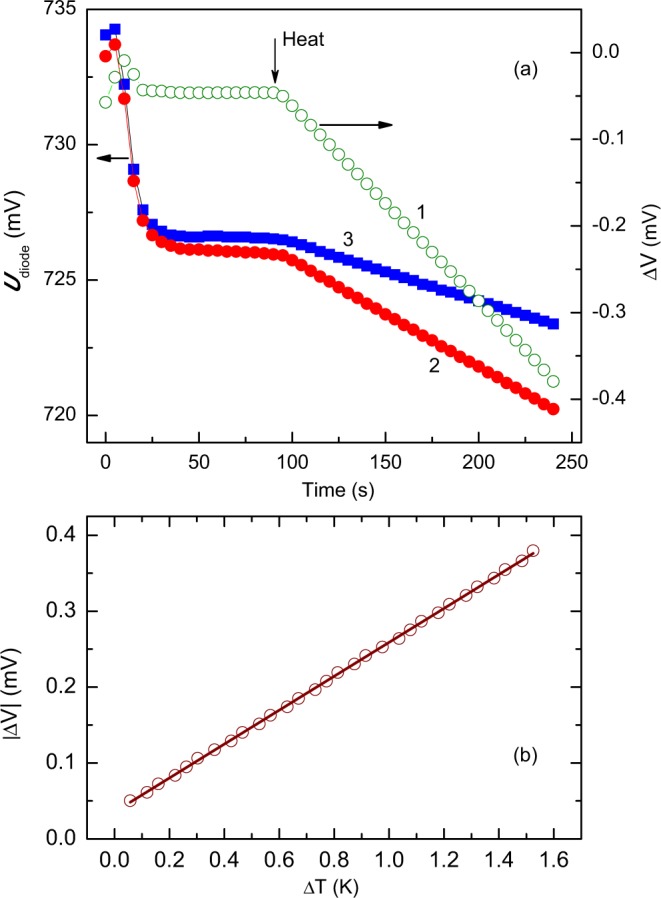
(**a**) Real-time monitoring of the temperature-sensing diode voltages (curves 2 and 3 correspond to diodes 2 and 3 in Fig. 3) after the cryostat temperature controller is set to 5° temperature increase at *t* = 0 and after the heater 1 in Fig. 3 is energised at *t* = 90 s. The diode voltages and Δ*V* (curve 1) are read with a 16 bit analog-to-digital converter (Analog Devices AD7792). (**b**) Voltage monitoring during Seebeck coefficient measurements in regular-QD sample at *T* = 243 K. Circles - experimental data. Line is a linear fit to the data yielding *S* = 0.223 mV/K.

In our experimental setup, the heater current *I*_*R*_ is determined by measuring an applied voltage, which varies as square root of time. In this case, both the electrical power *I*_*R*_^2^*R* and the temperatures sensed by diodes 2 and 3 increase with a linear dependence on time. This behaviour is exemplified in Fig. 4(a). At time *t* = 0, the controller of the cryostat temperature is set to a 5° larger temperature value, so the diode readings evolve in time, as shown by curves 2 and 3 in Fig. 4(a). Well after the readings saturate and the temperature is stabilized at the larger value of *T* (horizontal part of curves 2 and 3 at *t* ≥ 40 s in Fig. 4(a)) the heating is applied to the heater 1 in Fig. 3 at *t* = 90 s (arrow “Heat” in Fig. 4(a)).

When current is applied through the heater (1 in Fig. 3), there is a temperature rise behind the heater area, which yields the temperature difference Δ*T* between the sensors (2 and 3) sitting on the sample surface. Hence, curves 2 and 3 in Fig. 4(a) gradually diverge at time instants greater than that marked by arrow “Heat”. The resulting temperature difference Δ*T* increasing in time creates a thermoelectric voltage Δ*V* (curve 1 in Fig. 4(a)), which responses to the changes in Δ*T*. Figure 4(b) shows the linear fit to the measured Δ*V* vs Δ*T* data, which has a slope of 0.223 mV/K equal to the value of the Seebeck coefficient.

Further experimental details are given below in *Methods section*.

## Results and Discussion

**Figure 5:**
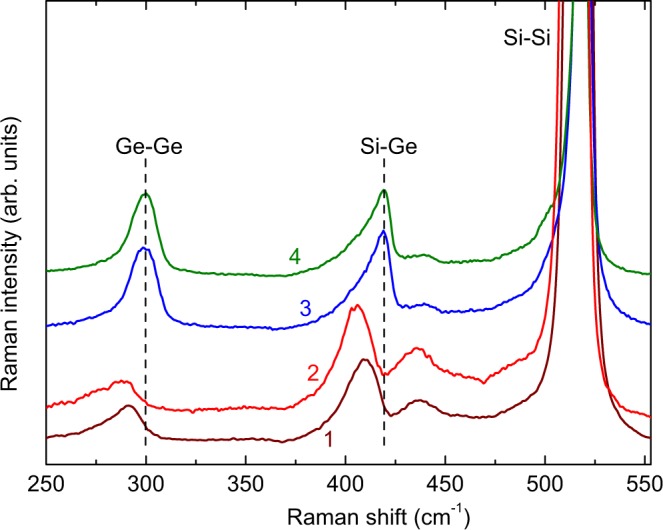
Raman spectra of samples L (1) and MDL (2), 2-fold (3) and 3-fold (4) coupled QD structures. Two dashed lines are used to guide the eye to show the different frequencies of the Ge-Ge and Si-Ge modes.

Raman spectra of our samples are shown in Fig. 5. Three Ge_*x*_Si_1−*x*_ alloy phonon modes are clearly seen, which correspond to Ge-Ge (at frequency *ω*_1_), Si-Ge (*ω*_2_) and Si-Si vibrations. It is known that the frequencies of the Ge-Ge and Si-Ge modes vary with the Ge fraction *x* and in-plane strain 〈*S*_∥_〉 as follows^40^:

1 $${\omega }_{1}=284+5x+12{x}^{2}+{b}_{1}\langle {S}_{\parallel }\rangle ,$$

2 $${\omega }_{2}=400+29x-95{x}^{2}+213{x}^{3}-170{x}^{4}+{b}_{2}\langle {S}_{\parallel }\rangle ,$$

where *b*_1_ and *b*_2_ are the phonon strain-shift coefficients for the Ge-Ge and Si-Ge modes, respectively. In order to obtain an estimate of the *x* and 〈*S*_∥_〉 values, we use *b*_1_ = −400 cm^−1^ and *b*_2_ = −575 cm^−1^ given by Tan *et al*.^41^ and Lin *et al*.^42^. The calculated values for these parameters are given in Table 1.

**Table 1:** The Ge-Ge and Si-Ge Raman frequencies obtained from spectra in Fig. 5 and the values of *x* and 〈*S*_∥_〉 calculated using Eqs 1 and 2.

Sample	*ω*_1_ (cm^−1^)	*ω*_2_ (cm^−1^)	*x*	〈*S*_∥_〉 (%)
L	291.7	410.3	0.34	−0.008
MDL	288.2	406.3	0.25	−0.003
2-fold CQDs	299.2	418.7	0.54	−0.020
3-fold CQDs	299.8	419.1	0.57	−0.021

It is seen that the concentration of Ge atoms in the CQDs layers is about 55%, showing that intermixing of Si and Ge occurs during the growth process, which is a common process in crystallization of Si/Ge multilayers^43,44^. It is also seen in Table 1 that in both 2-fold and 3-fold CQDs layers the values of *x* are very close to each other.

In order to decipher how the QD structure evolves during this process and provide supporting evidence for the intermixing, we performed a numerical study based on molecular dynamics (see Supplementary Video). From this simulation, the value *x* = 0.67 was obtained, which is in reasonable overall agreement with that obtained in Raman experiments.

**Figure 6:**
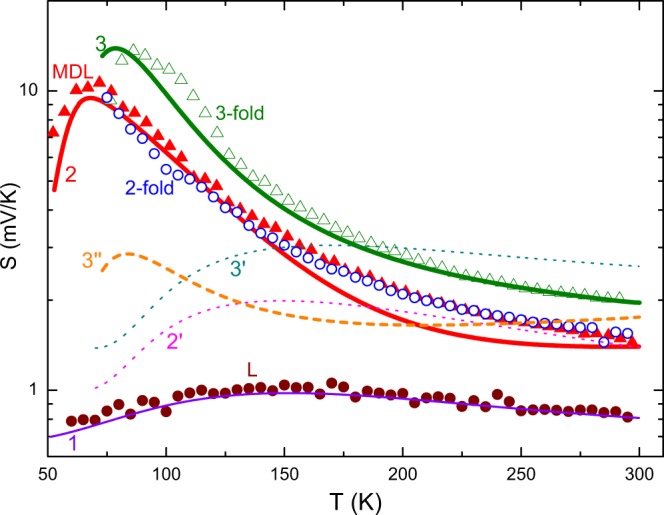
Temperature dependence of *S* for samples L, MDL, 2-fold and 3-fold coupled QD structures studied experimentally (data points) and theoretically (lines).

The measured temperature dependencies of the Seebeck coefficient for different samples are shown by the data points in Fig. 6. These data indicate that *S* drops with decreasing electrical resistivities of our samples (≈0.09 Ω × cm in CQDs, 0.04 Ω × cm in MDL, 0.03 Ω × cm in L), which follows the trend established in Fig. 1.

As stated in the Introduction, computations can be based on the Boltzmann transport equation. The comparison between experimental data of Fig. 6 and model predictions was carried out considering the contribution of the diffusive *S*_*d*_ and phonon drag *S*_*p*_ components into the Seebeck coefficient *S*. Based on the preceding literature survey, we employ the general calculation framework addressed by Mahan *et al*.^45^. The carrier diffusion term in *n*-type layers is

3 $${S}_{d}=\frac{1}{eT}[{E}_{c}-\mu +\frac{{I}_{2}}{{I}_{1}}{k}_{B}T].$$

Similarly for *p*-type layers

4 $${S}_{d}=\frac{1}{eT}[{E}_{c}+{E}_{g}+\mu +\frac{{I}_{2}}{{I}_{1}}{k}_{B}T],$$

where *e* is the electron charge, *E*_*c*_ is the conduction band minimum, *E*_*g*_ is the band gap, *μ* = *μ*(*T*) is the Fermi level, *k*_*B*_ is the Boltzman constant and the ratio *I*_1_/*I*_2_ is taken in the integral forms

$${I}_{1}={\int }_{0}^{\infty }\,{\xi }^{3/2}\tau (\xi ){e}^{-\xi }d\xi ,$$

$${I}_{2}={\int }_{0}^{\infty }\,{\xi }^{5/2}\tau (\xi ){e}^{-\xi }d\xi ,$$

where the variable $\xi =\varepsilon (\overrightarrow{k})/{k}_{B}T$ is the dimensionless energy $\varepsilon (\overrightarrow{k})$ of the carrier with the wave vector $\overrightarrow{k}$ and lifetime *τ*(*ξ*).

In sample L, we consider a uniformly doped single layer of Ge _0.34_Si _0.66_ and combine relevant scattering mechanisms to get total phonon (*τ*_*p*_) and electron (*τ*_*e*_) lifetimes. These can be numerically calculated in the framework described, for example, by Ziman^46^. Here, we include phonon-phonon normal and Umklapp processes, alloy scattering and boundary scattering of phonons at the inner boundaries for calculating *τ*_*p*_. The computed *τ*_*e*_ value takes into account different scattering centers such as impurities and phonons. These scattering mechanisms were combined using the Matthiessen’s rule.

The phonon lifetime *τ*_*p*_ is calculated by following a procedure given by Mahan *et al*.^45^. In our fitting, input parameters for silicon (not SiGe) are used, which are taken from ref. ^45^. To account for Ge_*x*_Si_1−*x*_ alloy, we replace isotope scattering by alloy scattering in Ge_*x*_Si_1−*x*_ with the value of *x* taken from Table 1. The electron lifetime *τ*_*e*_ is obtained for Ge_*x*_Si_1−*x*_ using the approach and input parameters described by Yi and Yu^47^. The model we use here also involves a set of additional physical parameters which are taken from the literature^45,47^. The solid line 1 in Fig. 6 represents the fitting result in sample L, exhibiting an excellent agreement with measurement data given by closed circles.

However, this approach cannot be straightforwardly applied to MDL and CQD samples, as illustrated by curves 2′ and 3′ in Fig. 6. It is seen that similarly modeled *S*(*T*) curves to include *τ*_*p*_ and *τ*_*e*_ exhibit a noticeable discrepancy between the expected (curves 2′ and 4′) and measured (closed and open triangles in Fig. 6, respectively) values of *S*. Based on the experimental results given in the Introduction, we may therefore conclude that in our MDL and CQD samples enhancement of the phonon scattering due to inner boundaries in the Ge_*x*_Si_1−*x*_ layer and Ge/Si/Ge stacks has to be taken into account.

Speaking qualitatively, the phonons preferentially move from the hot to cold sample side in the temperature gradient across them. This in turn forces the carriers to move in the same direction and this effect is gradually quenched with increasing *T* due to anharmonic interatomic forces^48^. This would increase *S* at low temperatures, as indeed observed in our *S*(*T*) experiments. It is seen in Fig. 6 that this rise in *S* is most pronounced in MDL and CQD samples (open circles and triangles). The decrease in *S* with further decreasing temperature can naturally be explained by the contribution of the boundary scattering of phonons^48,49^. As expected, increasing the number of embedded scatterers would make this effect more marked, which is also observed in Fig. 6 (open triangles compared with open circles).

**Figure 7:**
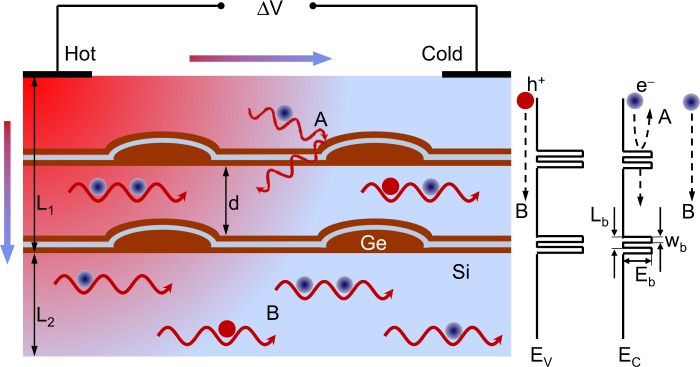
Schematics of phonon scattering processes from the 2-fold CQD inclusions (A) and the phonon drag (B) contribution to the thermoelectric voltage Δ*V*. Electron and hole are indicated by a circle and labeled *e*^−^ and *h*^+^, respectively. Horizontal and vertical arrows illustrate a heat flux from hot to cold parts of the sample. *L*_1_ and *L*_2_ indicate the thickness of the quantum dot and buffer Si layers, respectively. In our CQD samples, *L*_2_ = 50 nm and *L*_1_ ≈ 1.4 *μ*m in 2-fold and 1.8 *μ*m in 3-fold CQD structures. Computationally, a one-dimensional unit cell spans the buffer Si layer (*L*_2_) and 4 periods of CQD layers (*L*_1_ = 140 nm in 2-fold and 180 nm in 3-fold CQDs). *L*_*b*_ and *w*_*b*_ indicates the fitting parameters of the barriers generated by embedded CQDs (see Supplementary Fig. S4). High-energy electrons move above the energy barrier (process B on the right-hand side) while the ones with energies less than the barrier height are scattered back (process A). Conduction- and valence-band edges are marked as *E*_*c*_ and *E*_*v*_, respectively.

Therefore, following the procedure described above, we now consider two more relaxation processes for modeling *S*(*T*) curves in MDL and CQD samples, which involve the scattering from the inner interfaces in *τ*_*p*_ and the carrier filtering in *τ*_*e*_. In both samples, the boundary scattering is taken into account when calculating the phonon drag term as

5 $${S}_{p}=\frac{{k}_{B}d{D}_{a}^{2}{\hslash }^{2}{\theta }_{D}^{5/2}}{64\pi {\rho }_{0}e{a}_{B}^{6}{E}_{i}^{3}{T}^{5/2}}\frac{{I}_{3}}{{I}_{1}},$$

where *d* is the distance between boundaries and interfaces that scatter incident phonons (see Fig. 7), *D*_*a*_ is the deformation potential parameter, $\hslash$ is the Planck constant, *θ*_*D*_ is the Debye temperature, *ρ*_0_ is the sample density, *a*_*B*_ is the effective Bohr radius of the impurity, *E*_*i*_ is the binding energy of the impurity and

$${I}_{3}=\frac{d}{{\upsilon }_{L}}{\int }_{0}^{\infty }\,\tau (\xi ){e}^{-\xi }d\xi {\int }_{0}^{2k{a}_{B}}\,{y}^{4}{\tau }_{p}(y)\coth (\frac{y\hslash {\upsilon }_{L}}{2T{k}_{B}{a}_{B}})dy,$$

$$\frac{1}{{\tau }_{p}(y)}=\frac{{\upsilon }_{L}}{d}(\frac{1-\alpha }{1+\alpha })+\frac{{g}_{2}{{\rm{\Omega }}}_{0}{\upsilon }_{L}}{24\pi {a}_{B}^{4}}(1+2\frac{{\upsilon }_{L}^{3}}{{\upsilon }_{T}^{3}}){y}^{4}+\frac{{y}^{\delta }}{{\tau }_{p0}},$$

where *υ*_*L*_ and *υ*_*T*_ are the speeds of the longitudinal and transverse acoustic waves, respectively, *y* = *qa*_*B*_ is the phonon integration variable, *q* is the phonon wave number, *g*_2_ is the coupling coefficient for isotope scattering and Ω_0_ is the unit cell volume. The interface specularity parameter *α* is included in the first term of the phonon scattering rate *τ*_*p*_^−1^(*y*) as discussed elsewhere^47^. For the specular reflection *α* = 1, and for the totally diffusive scattering of phonons *α* = 0. Values of *δ* (ranging from 1.4 to 2.3) and *τ*_*p*0_ were found to be very temperature dependent^45^.

For handling the total Seebeck coefficient, we artificially divide each layer *d* in Fig. 7 into a certain number *N* of sublayers having a thickness of *d*_*i*_ (*i* = 1,2, ..., *N*). We choose here the thickness of the sublayer varying from 15 to 25 nm. Following a calculation similar to that of Bahk *et al*.^50^, the total measured value of *S* is the weighted averaging of the *mN* values developed in the sublayers with *m* being the number of repeated layers (*d* in Fig. 7) in the sample. The weights are written in proportion to the electrical conductivity *σ*_*i*_ of the *i*-th sublayer multiplied by the thickness *d*_*i*_ as

6 $$S=\frac{{\sum }_{i}\,({S}_{d,i}+{S}_{p,i}){\sigma }_{i}{d}_{i}}{{\sum }_{i}\,{\sigma }_{i}{d}_{i}},$$

where the subscript *i* denotes the constituent *S*_*d*_ and *S*_*p*_ coefficients for the *i*-th sublayer. The electrical conductivity is defined^47^ by

7 $${\sigma }_{i}=\frac{{e}^{2}{(2{m}_{i}^{\ast }{k}_{B}T)}^{3/2}}{3{\pi }^{2}{m}_{i}^{\ast }\hslash }{\int }_{0}^{\infty }\,\frac{{\tau }_{i}{\xi }_{i}^{3/2}{\exp }({\xi }_{i}-\mu )}{{[{\exp }({\xi }_{i}-\mu )]}^{2}}d{\xi }_{i}$$

where m^*^ is the effective mass of the carrier and *τ*_*i*_ is the total lifetime in the *i*-th sublayer. Using Matthiessen’s rule, *τ*_*i*_ was obtained from the lifetimes due to different scattering mechanisms discussed above.

The specularity parameter *α* and the value of *τ*_*p*0_ in the phonon scattering rate *τ*_*p*_^−1^(*y*) were adjusted to match the *S*(*T*) data from the experiment. Solid line 2 in Fig. 6 is the best-fit curve to the filled triangular experimental data points for sample MDL. It is seen that curve 2 gives a good fit at temperatures below ≈150 K and at about *T* = 300 K. The model accounting for the interface scattering (curve 2 in Fig. 6) produces much better agreement with the experimental data for temperatures ≤175 K than the model yielding curve 2′.

This is in marked contrast to what one observes for multifold Ge/Si/Ge CQD structures. Indeed, dashed line 3″ in Fig. 6, computed similar to curve 2 for MDL sample, shows discrepancy to the experimental values (open triangles), which are too large at low temperatures and are somewhat smaller at temperatures greater than ≈200 K. We, therefore, have considered a subsidiary contribution of the carrier filtering to explain the experimental data for CQDs shown by open circles and triangles in Fig. 6. These calculations show that the *S*(*T*) data obtained in our experiments can be properly modeled by filtering the carriers with energies less than the barrier height.

Here we use the schematics of the scattering and filtering effects described by Zhou *et al*.^30^. Schematic illustration of the interaction between phonons and CQD inclusions and phonon drag processes is shown in Fig. 7 by arrows A and B, respectively. The quantum dot layer with thickness *L*_1_ and thin buffer layer (*L*_2_), which are included into our calculation unit cell, are depicted in Fig. 7. In the type II SiGe/Si heterostructure, the band edge discontinuities have different signs for the conduction and valence bands^51^, as indicated in the right-hand side of Fig. 7. The interaction of electrons with CQDs (process A in Fig. 7) is modeled by the relaxation time *τ*_*bi*_, which is approximated by the interface scattering in the *i*-th sublayer. If the average energy of moving electrons is less than the barrier height *E*_*b*_, they bounce back from the barrier (A in the right-hand side of Fig. 7). If the energy of electrons or holes exceeds the barrier height *E*_*b*_, they pass through the boundary region, as shown by processes B in Fig. 7.

The quantity *τ*_*bi*_ = *λ*_*i*_/*υ*_*i*_, where ${\upsilon }_{i}=\sqrt{2{\varepsilon }_{i}/{m}_{i}^{\ast }}$ is the average velocity of the carriers with energy *ε*_*i*_ and *λ*_*i*_ is their mean-free paths^52^

8 $${\lambda }_{i}=\frac{T({\varepsilon }_{i})({L}_{b}-{w}_{b})}{1-T({\varepsilon }_{i})}$$

with *T*(*ε*_*i*_) the transmission probability for the carriers through a barrier.

We find that this method provides an adequate fit to the experimental *S*(*T*) data in CQD samples using only *L*_*b*_ and *w*_*b*_ as slightly adjustable parameters (aside from the ones used to fit the data in sample MDL). The model fit to the temperature-dependent Seebeck coefficient of 3-fold CQDs is shown by solid line 3 in Fig. 6. In all the temperature range, the agreement between the experimental values and those found from the fit to *S*(*T*) is excellent. In the case of 2-fold CQDs (open circles in Fig. 6), the discussed scattering effects are capable to consistently explain the experimental results, so that the the measured Seebeck coefficient and its temperature dependence can be fitted quite well by the computed curve (not shown in Fig. 6).

The values of the parameters used to generate the fits are *L*_*b*_ = 90 nm (3-fold CQDs), 100 nm (2-fold CQDs), *w*_*b*_ = 40 nm (3-fold CQDs), 60 nm (2-fold CQDs), *α* = 1.2 × 10^−2^ (3-fold CQDs), 2.3 × 10^−2^ (2-fold CQDs) and *E*_*b*_ = 0.37 eV in both samples (see Supplementary Fig. S4). Perhaps the most interesting result is that the specularity parameter *α* in 2-fold CQDs is roughly two times greater than the appropriate value observed in 3-fold CQDs, indicative of the fact that the interface is seen more rough in the latter sample with increased number of Si layers inserted into the quantum dot. It is most remarkable that the Seebeck coefficient corresponding to different CQD samples concomitantly increases with increasing the number of the inserted layers. Because the only difference between the 2-fold and 3-fold CQD samples is the one more Si layer inserted into the 3-fold quantum dot that leads to enhanced interface scattering of phonons, which, in turn, increases *S*. It is seen in Fig. 6 that the experimental *S*(*T*) data corresponding to the two CQD samples are nearly parallel to each other above ≈80 K (open circles and triangles), exhibiting increase in *S* of about 40% due to additional Si layer inserted into the dot.

**Figure 8:**
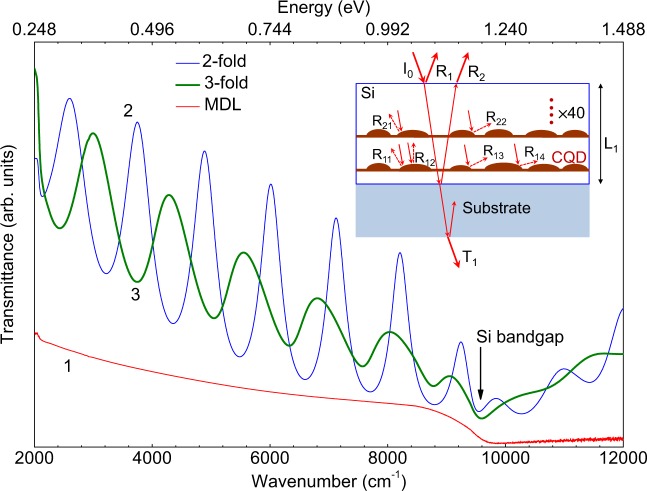
Room temperature IR transmittance spectra of sample MDL (1), 2-fold (2) and 3-fold (3) CQD structures. Inset: Ray diagram of the incident light (*I*_0_) within a CQD sample with the reflection from the front and rear surfaces and various interfaces. *R*_11_, *R*_12_, ..., *R*_22_ illustrate reflectivities from the inner boundaries.

Another test of this type, estimating the degree to which the interface scattering effects can be modified using composite quantum dots, is shown in Fig. 8. In this figure we plot the infrared (IR) transmittance spectra for MDL and CQD samples. In this experiment, the sample is illuminated from the front with an intensity of the incident light of *I*_0_ (inset in Fig. 8). The incident radiation is in part reflected at the layer top and bottom surfaces, as indicated by arrows *R*_1_ and *R*_2_, respectively, and transmitted through the sample (*T*_1_). Clear Fabry-Perot oscillations are observed in spectra 2 and 3 in Fig. 8 due to the interference between the partially reflected IR beams *R*_1_ and *R*_2_ shown in the inset of Fig. 8. However, these oscillations are not observable in sample MDL (spectrum 1 in Fig. 8). This is clearly in accord with the fact that the oscillations can occur if the layer thickness is comparable with the light wavelength due to the coherent multiple reflections^53^.

The second factor of observing the oscillations is a high enough optical transparency of the layered structure. This transparency is apparently affected by the presence of CQD scatterers between the two reflecting surfaces formed by the top and bottom interfaces of the deposited layer. Diffuse scattering of the light beam, which is highlighted by arrows *R*_11_, *R*_12_, …, *R*_22_ in the inset of Fig. 8, weakens the interference signals. Therefore, the amplitude of the transmitted light drops off very rapidly as the roughness of the inner interfaces is raised, as derived by Kan *et al*.^54^. Strictly speaking, specularity of an interface to an incident wave depends on the radiation wavelength relative to the roughness height and correlation length, as has been mentioned by Ogilvy^55^. Therefore, the scattering of electromagnetic and lattice waves should generally not be compared with each other. Meanwhile, it is remarkable that the quenching of the interference fringes observed in spectrum 3 of Fig. 8 compared with spectrum 2 and the increased *S* in 3-fold CQDs compared with that in 2-fold CQDs (open triangles and open circles in Fig. 6, respectively) both give a good indication of the increased interface roughness in 3-fold CQDs.

In conclusion, we have combined experiment and theory to deliver the first direct evidence that placing thin silicon layers inside the germanium quantum dot (multi-fold CQD material) can offer considerable enhancement of the Seebeck effect. We show that the Seebeck coefficient gets enhanced up to ≈40% in the 3-fold CQD material compared with the 2-fold CQDs. We present a numerical model to faithfully account for this enhancement, which relates the enlarged Seebeck coefficient to efficient scattering of phonons by inner boundaries and the carrier filtering by the CQD inclusions. These composite Ge/Si composite quantum dots can be competitive for the applications related to on-chip temperature control through micro-refrigeration and autonomous renewable energy systems. Moreover, we anticipate that the phonon scattering techniques developed here could significantly augment the thermoelectric performance of Ge/Si materials through further optimization of the layer stacks inside the quantum dot and of the dopant concentrations.

## Methods

### Sample characterization

Cross-sections of the microstructures were observed on a JEOL JEM-2100 transmission electron microscope (XTEM) operating at 200 kV. Raman spectra measurements were performed at room temperature with a 514.5-nm line of an Ar^+^ laser using a Horiba Jobin-Yvon T64000 spectrometer. The laser light was focused onto the sample surface to a spot of 0.7 *μ*m in diameter using Olympus objective (50×, NA = 0.5). Raman measurements were performed in the *z*(*x*, *y*)$\bar{z}$ scattering geometry, where *x*, *y*, and *z* correspond to the crystallographic directions [100], [010], and [001] of Si, respectively. This Raman geometry is allowed for scattering by LO phonons in Ge and Si and forbidden for two-phonon scattering by TA phonons of Si substrate, which thus allowed to avoid complications in the interpretation of Raman spectra^56^. Room temperature transmission FTIR spectra in the 2000–12000 cm^−1^ wavenumber range were obtained at normal incidence using Bruker Vertex 70V FTIR spectrometer with a Globar source. For each spectrum, 64 scans were averaged with a spectral resolution of 1 cm^−1^. The layer resistance was measured employing the transmission line measurement technique^57^.

### Simulations

Numerical analysis of the integrals *I*_1_, *I*_2_ and *I*_3_ in Eqs (3), (4) and (5) was performed using the extended trapezoidal rule and the Fortran code available on pp. 1052–1056 in the book of Press *et al*.^58^. Supplementary Video clip was created using the open visualization tool OVITO. The program is freely available under an open source license at https://ovito.org.

## Supplementary information

Supplementary Video Legend

Supplementary Figs. S1-S4
