# Supplementary material for: Enhancing the Seebeck effect in Ge/Si through the combination of interfacial design features
Source: Sci Rep. 2019 Nov 8;9:16335. doi: 10.1038/s41598-019-52654-z (PMC6841935; doi:10.1038/s41598-019-52654-z)
Supplement: Supplementary file 2 — Supplementary Figs. S1-S4 [file 41598_2019_52654_MOESM2_ESM.pdf]

Supplementary Information

Enhancing the Seebeck effect in Ge/Si through the combination of interfacial design features

Andriy Nadtochiy[1], Vasyl Kuryliuk[1], Viktor Strelchuk[2], Oleg Korotchenkov[1,*], Pei-Wen Li[3], Sheng-Wei Lee[4]

[1] Taras Shevchenko Kyiv National University, Department of Physics, Kyiv 01601, Ukraine
[2] V.E. Lashkaryov Institute of Semiconductor Physics, National Academy of Sciences of Ukraine, Kyiv 03028, Ukraine
[3] National Chiao-Tung University, Department of Electronic Engineering, Hsin-Chu 30010, Taiwan, Republic of China
[4] National Central University, Institute of Materials Science and Engineering, Jhongli 32001, Taiwan, Republic of China

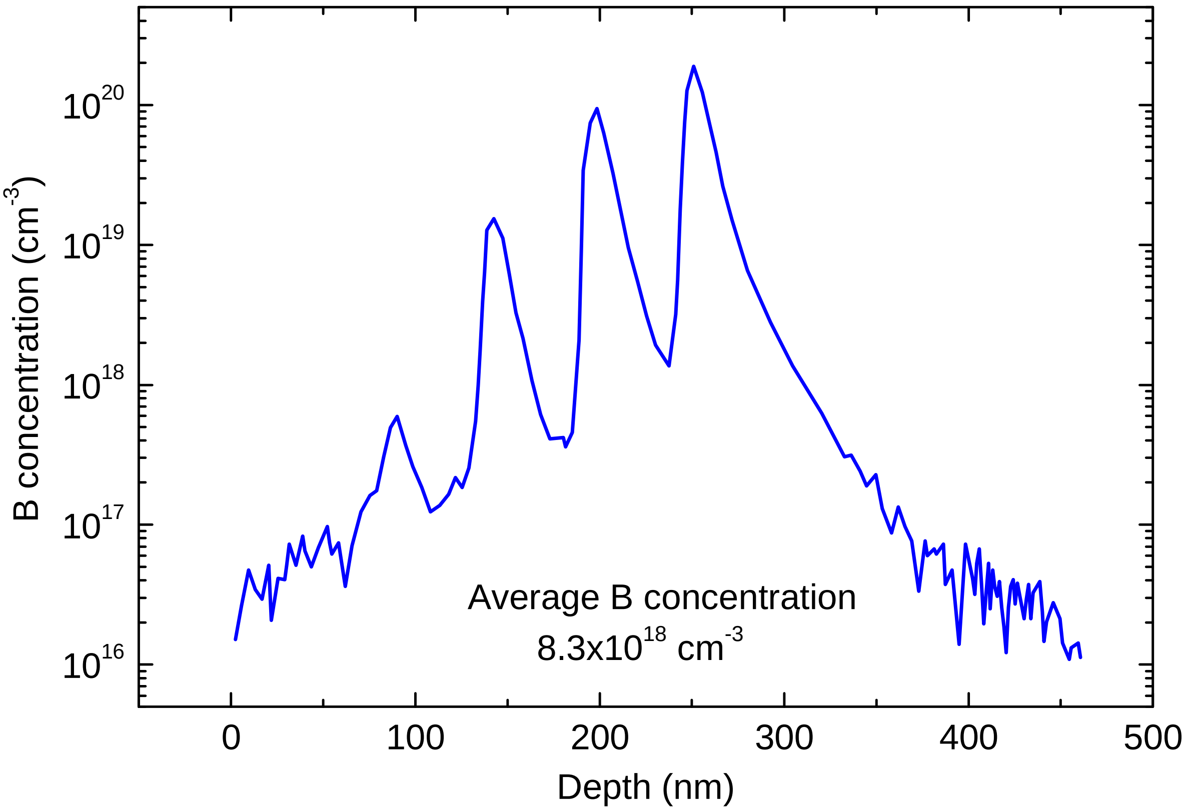

Supplementary Fig. S1. Depth profile of B concentration in four layers of B-doped epitaxial Ge_xSi_{1-x} film measured by SIMS. The atomic layer doping peaks are formed at different B_2H_6 partial pressures.

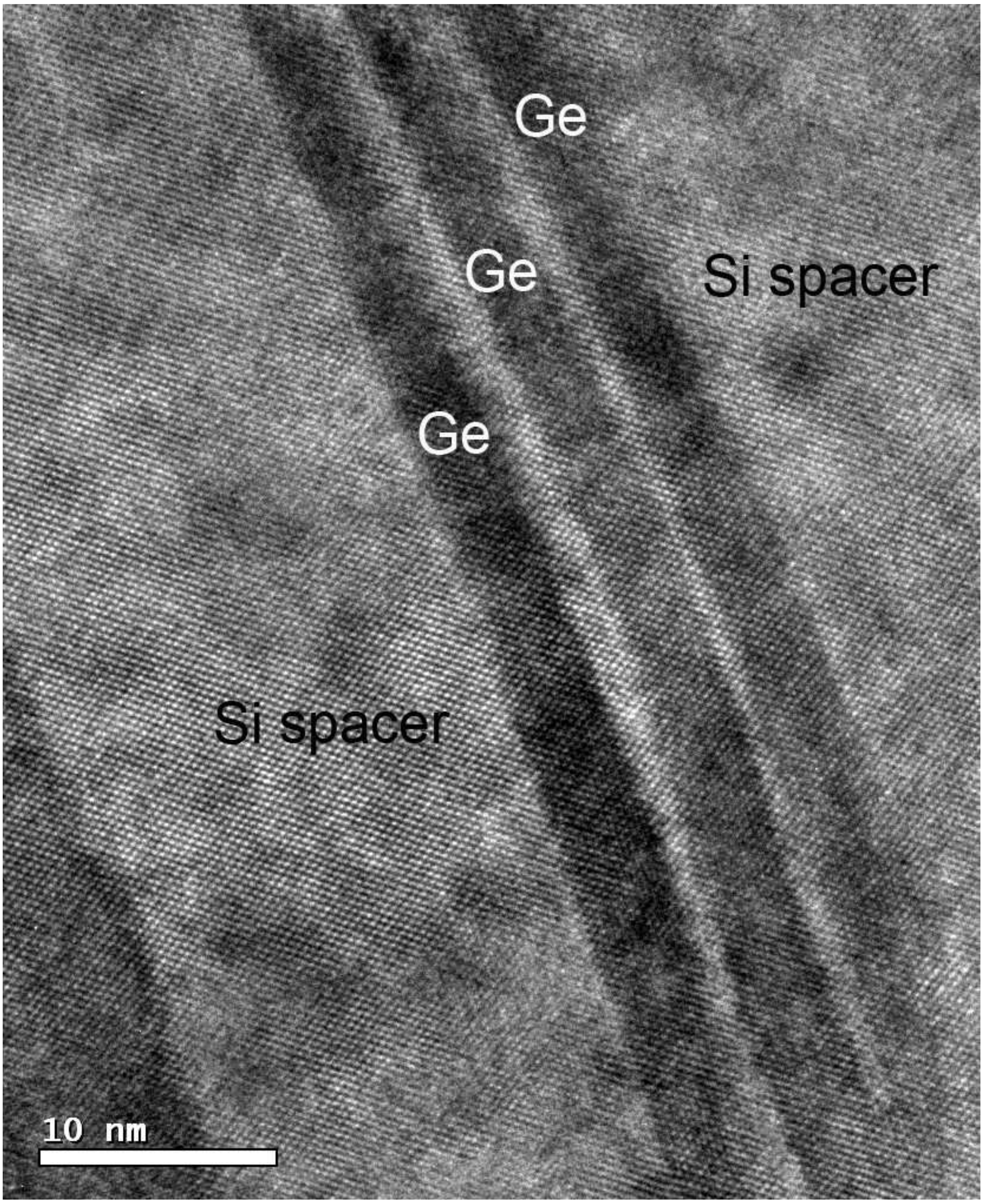

Supplementary Fig. S2. High-resolution XTEM micrograph showing a layer sequence inside a 3-fold-CQD (dark regions – Ge, light grey regions – Si).

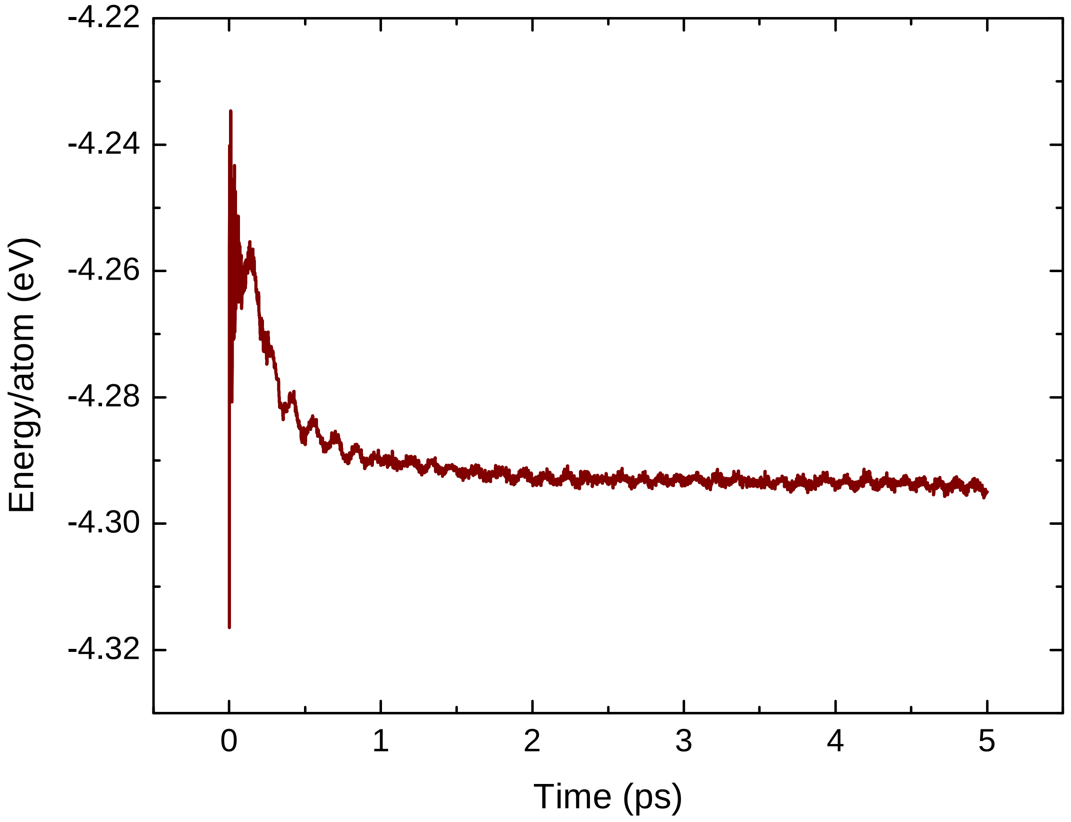

Supplementary Fig. S3. Calculated time evolution of the energy per atom of the Si matrix – Ge dot system (see Supplementary Video Legend).

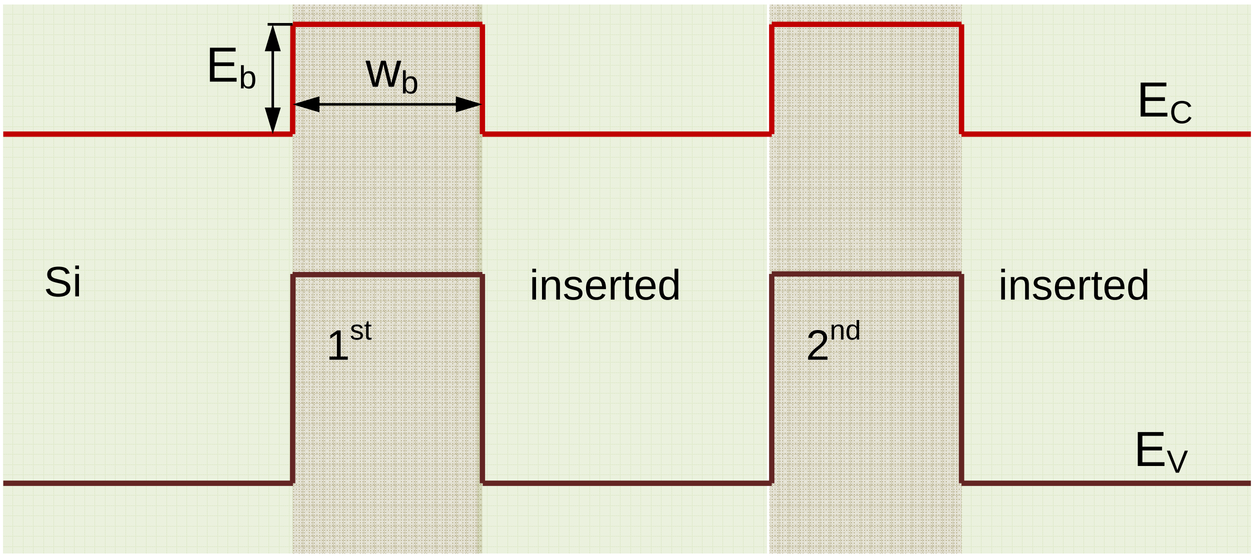

Supplementary Fig. S4. Schematic of two Ge layers in the CQD separated by Si layers inserted into the CQD, which are limited by rectangular potential barriers with the height E_b = 0.37 eV determined by the potential step at the Si/Ge interface [1]. The conduction (E_C) and valence band (E_V) edges are represented by thick solid lines. w_b indicates the dot barrier width (Fig. 7 in the main text).

Supplementary Video Legend

Supplementary Video clip illustrates the time evolution of the Ge (blue circles) and Si (red circles) atomic positions during 5 ps with the step of 2 fs after the Ge triangular dot is placed into the Si matrix at time moment t = 0. The temperature is 600°C. The Stillinger-Weber potential [2] is employed. The time evolution of the energy per atom of the Si matrix – Ge dot system, exemplified in Supplementary Fig. S3, demonstrates that the system has been settled down to a stable low energy state during 5 ps. This indicates that the Ge fraction in Ge_xSi_{1-x} saturates at a value of x = 67% discussed in the main text. Simulation details can be found in Ref. [3].

Supplementary References

1. Van de Walle, C. G. Band lineups and deformation potentials in the model-solid theory. *Phys. Rev. B* **39**, 1871–1883 (1989).
2. Stillinger, F.H. & Weber, T.A. Computer simulation of local order in condensed phases of silicon, *Phys. Rev. B* **31** 5262–5271 (1985).
3. Kuryliuk, V.V. & Korotchenkov, O.A. Atomistic simulation of the thermal conductivity in amorphous SiO_2 matrix/Ge nanocrystal composites. *Physica E* **88**, 228–236 (2017).
